# Therapeutic efficacy of glucocorticoids, ATP, and antithyroid drugs in acute thyrotoxic myopathy: evaluation using the acute thyrotoxic myopathy symptom score

**DOI:** 10.3389/fendo.2026.1769220

**Published:** 2026-05-21

**Authors:** Qiaoli Li, Xuelan Chen, Zien Huang, Yun Zhang, Yaqi Kuang, Chunjiao Wu, Shien Fu, Tingliang Wang, Yuping Liu, Zhenxing Huang, Haiyan Yang, Jing Xian, Li Li, Jia Zhou, Xinghuan Liang, Yingfen Qin, Zuojie Luo

**Affiliations:** 1 Department of Endocrinology, The First Affiliated Hospital of Guangxi Medical University, Nanning, China; 2 Department of Endocrinology, Affiliated Hengyang Hospital of Hunan Normal University and Hengyang Central Hospital, Hengyang, Hunan, China; 3 Department of Pathology, The People’s Hospital of Guangxi Zhuang Autonomous Region and Guangxi Academy of Medical Sciences, Nanning, China; 4 Department of Endocrinology and Metabolism, Binzhou Medical University Hospital, Binzhou, China

**Keywords:** acute thyrotoxic myopathy, glucocorticoids, adenosine triphosphate, antithyroid drugs, acute thyrotoxic myopathy symptom score

## Abstract

**Background:**

Acute thyrotoxic myopathy (ATM) is a relatively rare and severe complication of hyperthyroidism, mainly reported in individual cases and lacking standardized treatment protocols.

**Objective:**

This study evaluated the efficacy of glucocorticoids (GCs), adenosine triphosphate (ATP), and antithyroid drugs (ATDs) in treating ATM to guide clinical management.

**Methods:**

We retrospectively analyzed 42 ATM patients treated with GCs, ATP, and ATDs. The Acute Thyrotoxic Myopathy Symptom Score (ATMSS) was applied to evaluate efficacy at baseline (T0), day 7 (T7), and day 28 (T28) of treatment. Binary logistic regression was used to identify predictors of therapeutic efficacy, examining the impact of pre-treatment biochemical indicators and ATMSS on symptom relief (R) at T7 and complete remission (CR) at T28.

**Result:**

ATMSS demonstrated significant improvement at both T7 and T28 (*P* < 0.001). R and CR rates differed among symptoms, with nasal reflux, dysphagia, and dyspnea showing markedly higher rates than dysarthria, gag reflex, and muscle weakness (T7: χ² = 30.89, T28: χ² = 36.71, both *P* < 0.001). Binary logistic regression analysis indicated that higher baseline dysarthria scores were associated with lower R rates at T7 (*OR* = 0.216, 95% *CI* = 0.056–0.836, *P* = 0.027), while more severe muscle weakness at T7 predicted lower CR at T28 (*OR* = 0.564, 95% *CI* = 0.323–0.984, *P* = 0.044).

**Conclusion:**

The combination of GCs, ATP, and ATDs effectively alleviated the ATMSS in the short (7day) and medium (28day) term. However, recovery rates differ among symptoms. Specifically, dysarthria at baseline and muscle weakness at T7 showed significant predictive value for treatment outcomes.

## 1 Introduction

Acute thyrotoxic myopathy (ATM) is a serious and relatively rare neuromuscular complication of hyperthyroidism (1). Beyond the hypermetabolic symptoms of hyperthyroidism, ATM is characterized by manifestations of bulbar palsy, including nasal reflux, dysphagia, hoarseness, pharyngeal reflex impairment, and dyspnea (2). If the disease is not identified early and actively treated, the risk of aspiration pneumonia and acute respiratory failure will increase significantly. Currently, most knowledge of ATM is derived from individual case reports. The resultant limited evidence contributes to insufficient disease recognition among clinicians, often leading to underdiagnosis, delayed intervention, and an increased risk of life-threatening complications. Therefore, enhancing research efforts and improving clinical awareness of this disease are crucial.

The absence of universal guidelines for ATM treatment leads to a reliance on empirical regimens from case reports. While some experts recommend managing ATM as a thyroid storm (3), other cases are treated symptomatically with antithyroid drugs (ATDs) and beta-blockers (4, 5). Although often ultimately effective, conventional treatment typically results in a slow neuromuscular recovery. Weeks of ATDs treatment only begin to show improvement in symptoms of bulbar paralysis, such as swallowing difficulties and severe muscle weakness (6). It leads to prolonged hospitalization and an increased risk of disease progression. Case reports indicate that ATM can progress to aspiration pneumonia even during conventional treatment with ATDs and beta-blockers, with subsequent control achieved by adding corticosteroid and antibiotic therapy (3, 7). This suggests that conventional ATDs treatment may not be sufficient to block the pathophysiological mechanisms of acute neuromuscular injury. Previous research indicates that Thyrotropin Receptor Antibody (TRAb) mediates immune-inflammatory responses in neural cells (8), while elevated thyroid hormone (TH) levels induce mitochondrial dysfunction and energy deficits (9, 10). Given the pivotal role of high TRAb and TH levels in the pathogenesis of ATM, we propose an intensified treatment strategy comprising Glucocorticoids (GCs), adenosine triphosphate (ATP), and ATDs. This regimen is designed to concurrently control hyperthyroidism, suppress immune-mediated damage, and directly supplement cellular energy, thereby accelerating clinical symptom resolution. Our initial clinical experience provides supportive evidence for this hypothesis.

The objective of this study is to systematically assess the therapeutic efficacy of a regimen combining GCs, ATP, and ATDs over the short and medium term in ATM. In the past, there was a lack of quantifiable tools for evaluating the efficacy of ATM. For the first time, we used an original ATM Symptom Score (ATMSS) (11) to quantitatively evaluate the improvement of bulbar paralysis symptoms in patients on T7 and T28. This work is expected to establish a theoretical foundation for optimizing future clinical strategies for ATM.

## 2 Materials and methods

### 2.1 Research design

This study was a single-center, retrospective cohort study.

### 2.2 Patient population

We retrospectively analyzed the medical records of ATM patients hospitalized in the Department of Endocrinology of the First Affiliated Hospital of Guangxi Medical University from January 2022 to June 2025.

#### 2.2.1 Inclusion criteria

Patients who met all the following criteria were included in the study:

1. Clear diagnosis of hyperthyroidism: meeting the criteria of the Chinese Guidelines for the Diagnosis and Treatment of Thyroid Diseases, namely elevated levels of free thyroxine (FT4) and/or free triiodothyronine (FT3), decreased serum thyroid stimulating hormone (TSH) levels, and positive TRAb results.
2. Symptoms of bulbar paralysis, including newly developed choking cough, dysphagia, dysarthria, dyspnea, and abnormal pharyngeal reflex.
3. ATMSS not less than 4.5 points. The score is used to objectively quantify the severity of ATM bulbar paralysis and muscle weakness symptoms (11).

#### ​​2.2.2 Exclusion criteria

Patients with any of the following conditions were excluded:

1. Simple chronic thyroid myopathy (CTM) or other types of myopathies such as hypokalemia, hypokalemic periodic paralysis, or myasthenia gravis.
2. Patients with subacute thyroiditis were excluded.
3. Local organic diseases in the throat that can affect pronunciation, such as chronic pharyngitis, and vocal cord polyps.
4. Patients with psychological disorders such as anxiety, whose symptoms may be confused with those of myopathy.
5. Contraindications to glucocorticoid treatment (such as active hepatitis, severe gastrointestinal disease, infection, uncontrolled diabetes and osteoporosis).

### 2.3 Treatment plan

Reflecting real-world clinical practice, a retrospective analysis summarizes the combination therapies received by the patients as follows:

1. ATDs: To rapidly control thyroid toxicity, patients were administered methimazole or propylthiouracil based on their individualized conditions. Within the study cohort, the actual initial dosage range predominantly utilized by the treating physicians was 20–30 mg/d for methimazole or 200–300 mg/d for propylthiouracil.
2. GCs: Glucocorticoid administration followed an individualized, symptom-driven tapering strategy. A representative regimen during the hospitalization phase consisted of intravenous hydrocortisone (100mg/d Day1-2, 75mg/d Day3-4, 50mg/d Day5-6) or equivalent methylprednisolone was administered, followed by sequential treatment with oral hydrocortisone tablets (25mg/d Day7-13, 10mg/d Day14-20, 5mg/d Day21-28) or equivalent methylprednisolone tablets. The specific GC formulation and the exact tapering pace were adjusted according to the patient’s clinical response, tolerance, and hospital drug availability. For critically ill patients, the initial dose and length of hospital stay could be increased as appropriate. After 28 days, the decision to stop glucocorticoid treatment was evaluated based on patients’ condition.
3. ATP: Administer adenosine triphosphate disodium injection (40 mg/d) was administered intravenously for at least 5 days.
4. All patients received routine beta-blockers (such as propranolol) and symptomatic supportive treatment as needed according to their condition.

### 2.4 Efficacy evaluation

#### 2.4.1 Evaluation tools

ATMSS was used for evaluation, which includes six components: nasal reflux, dysphagia, dysarthria, dyspnea, gag reflex, and muscle weakness. Except for the gag reflex, which has a score range of 0–2 points. The score range for each evaluation item is 0–8 points, and the total score range is 0–42 points. The higher the score, the more severe the condition.

#### 2.4.2 Evaluation time point

At T0, T7, and T28, two trained physicians will independently evaluate the results. If the ATMSS is inconsistent, consensus will be reached through discussion.

#### 2.4.3 Grading criteria for therapeutic efficacy

1. Based on the reduction rate of ATMSS from baseline at treatment time points T7 and T28, the therapeutic efficacy is categorized into four grades as follows: Complete Remission (CR): The reduction rate of ATMSS reaches 100%, indicating all symptoms disappeared and the score has returned to zero. Significant Remission (MR): The reduction rate of ATMSS ranges from 75% to less than 100%. Remission (R): The reduction rate of ATMSS ranges from 50% to less than 75%. Poor Response (PR): The reduction rate of ATMSS is below 50%.
2. The therapeutic effect at T7 is divided into two grades: Remission (R) and Non-Remission (NR). A reduction rate of ATMSS equal to or greater than 50% is classified as R, while a reduction rate below 50% is rated as NR. Similarly, the therapeutic effect at T28 is divided into two grades: Complete Response (CR) and Incomplete Response (ICR). A reduction rate of ATMSS equal to 100% indicates CR, while a reduction rate below 100% indicates ICR.

### 2.5 Statistical analysis

Statistical analyses were conducted using SPSS Statistics 23.0. Normally distributed continuous data are expressed as mean ± standard deviation (x ± s), non-normally distributed data as median (interquartile range) [M (IQR1, IQR3)], and categorical data as number of cases (percentage) [n (%)].

Treatment efficacy was assessed by comparing ATMSS at T0, T7, and T28 using a Generalized Estimating Equation (GEE) model. The Wilcoxon signed-rank test was applied to analyze changes in thyroid function parameters from T0 to T28 and to evaluate efficacy ratings at T7 and T28. Differences in response rates (R at T7 and CR at T28) among symptoms were examined using the chi-square test or Fisher’s exact test. Predictors of treatment response (R at T7 and CR at T28) were identified through binary logistic regression analysis.

## 3 Results

### 3.1 Baseline characteristics of patients

A total of 42 patients with ATM were included in the study, comprising 6 males (14.29%) and 36 females (85.71%). These patients were predominantly young and middle-aged adults, with mostly normal or mildly altered body mass index (BMI). Blood biochemistry tests showed elevated FT3, FT4 and thyroid antibody levels. Additionally, serum potassium levels were normal. The baseline characteristics of the patients are summarized in **Table 1**.

**Table 1:** Baseline characteristics and blood biochemistry results in patients with ATM.

ATM(N=42)	n (%)/x ± s/M (IQR1,IQR3)
Gender	
Male	6 (14.29%)
Female	36 (85.71%)
Age (y)	32.5 ± 14.48
BMI (kg/m^2^)	19.72 ± 3.02
FT3 (pmol/L)	32.88 (18.16,46.08)
FT4 (pmol/L)	66.23 (42.93,77.23)
TSH (mIU/l)	0.01 (0.01,0.01)
TT3 (nmol/L)	8.73 ± 3.33
TT4 (nmol/L)	269.89 (225.38,308.93)
TGAb (%)	36.00 (9.71,63.53)
TRAb (IU/L)	21.94 (9.27,35.95)
TPOAb (IU/mL)	723.00 (279.80,1000.00)
Serum potassium (mmol/L)	4.02 ± 0.39

### 3.2 Changes in serum thyroid function levels in ATM patients before and after treatment

After 28 days of treatment, FT3 and FT4 levels in 42 ATM patients decreased significantly relative to baseline, with 20 patients showing a decrease in TRAb levels compared to before treatment. TSH levels increased compared to pre-treatment levels, as shown in **Table 2**.

**Table 2:** Comparison of serum thyroid function and TRAb levels between at T0 and T28 in patients with ATM.

Parameters	Before treatment	After treatment (T28)	*Z* value	*P* value
FT3 (pmol/L)	32.88 (18.16,46.08)	9.24 (5.73,15.33)	-5.094	0.000
FT4 (pmol/L)	66.23 (42.93,77.23)	21.94 (14.09,28.90)	-4.801	0.000
TSH (mIU/l)	0.01 (0.01,0.01)	0.01 (0.01,0.21)	-3.952	0.000
TRAb (IU/L)	23.37 (10.02,37.00)	16.15 (3.09,36.63)	-2.435	0.015

### 3.3 Efficacy analysis of GCs, ATP and ATDs in the treatment of ATM

**Figure 1:**
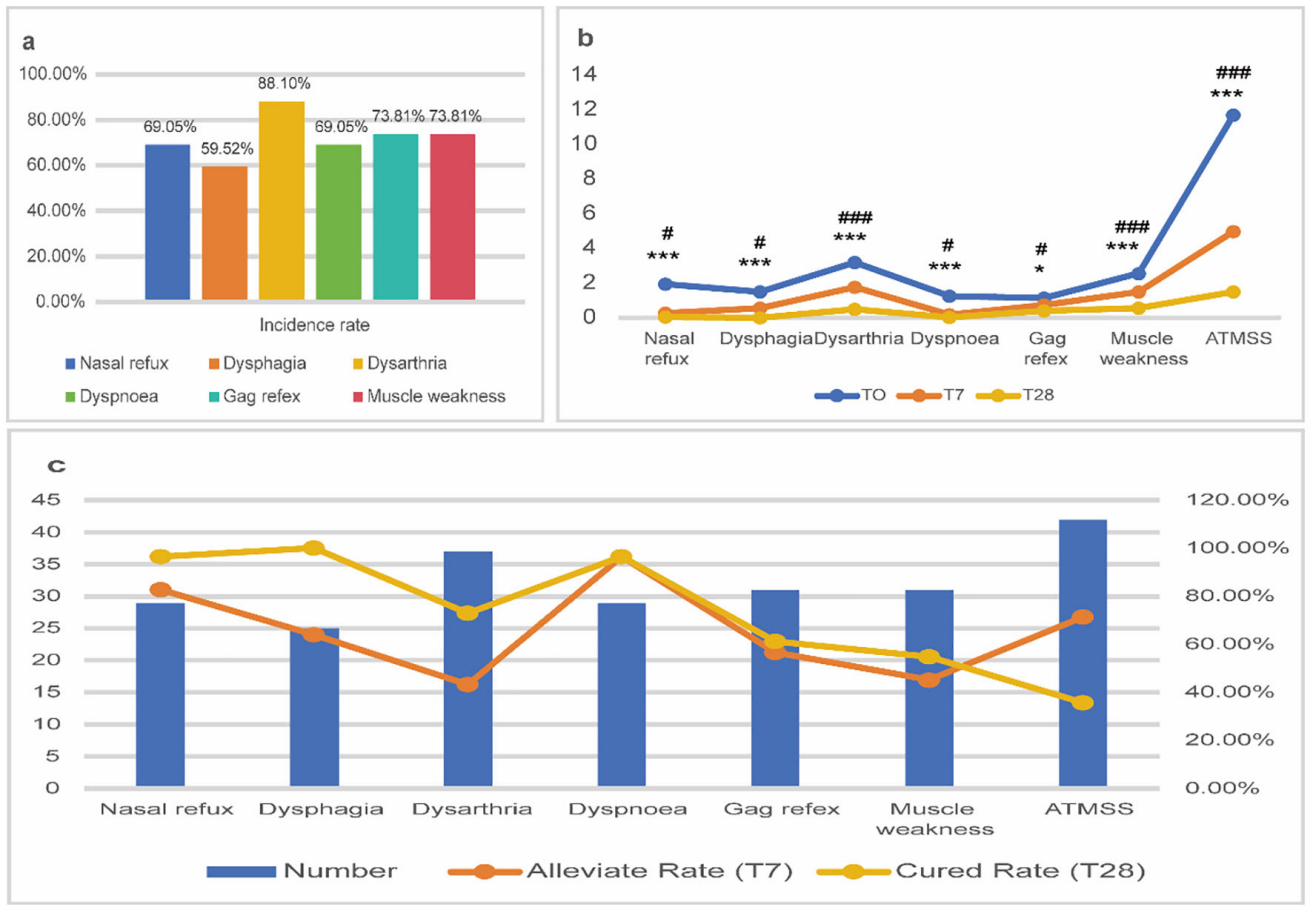
Efficacy assessment of combined therapy in patients with ATM. **(a)** The incidence rates of various symptoms in patients with ATM; **(b)** ATMSS before and after combined treatment. **(c)** Symptom relief rate at T7 and symptom cure rate at T28 in patients with ATM. **p<0.05, **p<0.01, ***p<0.001, T7 compared with T0; #p<0.05, ##p<0.01, ###p<0.001, T28 compared with T7.

All patients were hospitalized for more than 7 days. During this period, they were treated with GCs, ATP, and ATDs. After 7 days, patients were switched to treatment with oral GCs and ATDs, these medications were gradually reduced or discontinued depending on the patients’ clinical response. The results from the generalized linear model indicate that at T0, T7, and T28, all symptoms and the total ATMSS showed a statistically significant decrease (*P* < 0.001). These findings demonstrate that the combination therapy effectively alleviates multiple ATM symptoms in the short and medium term. The results are shown in **Table 3** and **Figure 1**.

**Table 3:** Comparison of symptom scores before and after combined treatment in patients with ATM.

Symptoms	Treatment time	Mean (SD)	*β* (95% CI)	*P* value
Nasal refux	T0	1.93 (0.30)		
	T7	0.26 (0.09)	1.67 (1.00,2.33)	0.000
	T28	0.05 (0.05)	1.88 (1.20,2.57)	0.000
Dysphagia	T0	1.50 (0.24)		
	T7	0.55 (0.14)	0.95 (0.54,1.36)	0.000
	T28	0.00 (0.00)	1.50 (0.94,2.06)	0.000
Dysarthria	T0	3.19 (0.28)		
	T7	1.76 (0.22)	1.43 (0.99,1.86)	0.000
	T28	0.48 (0.13)	2.71 (2.14,3.29)	0.000
Dyspnea	T0	1.24 (0.17)		
	T7	0.17 (0.08)	1.07 (0.63,1.51)	0.000
	T28	0.02 (0.02)	1.21 (0.81,1.62)	0.000
Gag reflex	T0	1.14 (0.12)		
	T7	0.74 (0.11)	0.40 (0.12,0.68)	0.000
	T28	0.40 (0.09)	0.74 (0.42,1.06)	0.000
Muscle weakness	T0	2.55 (0.34)		
	T7	1.50 (0.25)	1.05 (0.58,1.51)	0.000
	T28	0.55 (0.11)	2.00 (1.31,2.69)	0.000
ATMSS	T0	11.69 (0.73)		
	T7	4.98 (0.42)	6.71 (5.58,7.87)	0.000
	T28	1.50 (0.24)	10.19 (8.64,11.74)	0.000

The Wilcoxon signed-rank test was further employed to evaluate the relief of individual symptoms and the overall ATMSS grade at T7 and T28. At T7, significant relief was observed in nasal regurgitation, dysphagia, and dyspnea, whereas the improvement in dysarthria, gag reflex, and muscle weakness was relatively less pronounced. By T28, however, most symptom scores showed significant further improvement compared to T7 (*P* < 0.05), indicating continued symptomatic recovery during the later phase of treatment. These results underscore the sustained efficacy of the combination therapy in alleviating ATM symptoms over short- and medium-term follow-up, although the pace of recovery varied across symptoms, as summarized in **Table 4** and **Figure 1**.

**Table 4:** Comparison of symptom relief levels before and after combined treatment in patients with ATM.

Symptoms	After treatment (T7)	After treatment (T28)	*Z* value	*P* value
Nasal refux	1 (1,2)	1 (1,1)	-2.460	0.014
Dysphagia	1 (1,4)	1 (1,1)	-3.217	0.001
Dysarthria	4 (3,4)	1 (1,2.5)	-4.212	0.000
Dyspnea	1 (1,1)	1 (1,1)	-1.732	0.083
Gag reflex	3 (1,4)	1 (1,3)	-2.607	0.009
Muscle weakness	4 (3,4)	2 (1,3)	-3.985	0.000
ATMSS	3 (3,4)	2 (1,2)	-5.219	0.000

The efficacy level is defined as follows: 1 indicates complete remission; 2 indicates significant remission; 3 indicates partial remission; 4 indicates poor efficacy.

Among the 42 ATM patients, the most prevalent symptoms were dysarthria (88.10%), followed by abnormal gag reflex and muscle weakness (73.81%), nasal reflux (69.05%), dyspnea (69.05%), and dysphagia (59.52%, **Figure 1**). Response rates (relief at T7 and cure at T28) differed significantly across symptoms (T7: χ²=30.89, P<0.001; T28: χ²=36.71, P<0.001), with nasal reflux, dysphagia, and dyspnea responding more favorably than dysarthria, pharyngeal reflex impairment, and muscle weakness. These results demonstrate that treatment efficacy is symptom-dependent, revealing marked heterogeneity in recovery among patients (**Table 5**).

**Table 5:** Comparison of symptom relief rates before and after combined treatment in patients with ATM.

Efficacy	Nasal refux	Dysphagia	Dysarthria	Dyspnea	Gag reflex	Muscle weakness	*X^2^*	*P*
After treatment (T7)								
Alleviate	24 (82.76%)	16 (64.00%)	16 (43.24%)	28 (96.55%)	16 (56.61%)	14 (45.16%)		
Not alleviate	5 (17.24%)	9 (36.00%)	21 (56.76%)	5 (17.24%)	15 (48.39%)	17 (54.84%)	30.89	0.000
After treatment (T28)								
Cured	28 (96.55%)	25 (100.00%)	27 (72.97%)	28 (96.55%)	19 (61.29%)	17 (54.84%)		
Not cured	1 (3.45%)	0 (0.00%)	10 (27.03%)	1 (3.48%)	12 (38.71%)	14 (45.16%)	36.71	0.000

### 3.4 Factors affecting the therapeutic efficacy of T7 and T28 in ATM patients

Separate binary logistic regression models were constructed to evaluate factors affecting remission at T7 and complete relief at T28. In the model for T7 remission, a higher pre-treatment dysarthria score was a significant independent risk factor (OR = 0.216, 95% CI: 0.056–0.836, P = 0.027). In the model for T28 complete relief, a higher muscle weakness score at T7 was an independent predictor of outcome (OR = 0.564, 95% CI: 0.323–0.984, P = 0.044). These results demonstrate that the severity of specific symptoms, particularly dysarthria at baseline and muscle weakness at T7, has predictive value for treatment efficacy (**Table 6**).

**Table 6:** Factors impacting the treatment outcomes of T7 and T28 in patients with ATM.

Dependent variable	Independent variable	*P value*	*OR(95% CI)*	
Alleviate (T7)	FT3	0.103	1.119(0.978,1.280)	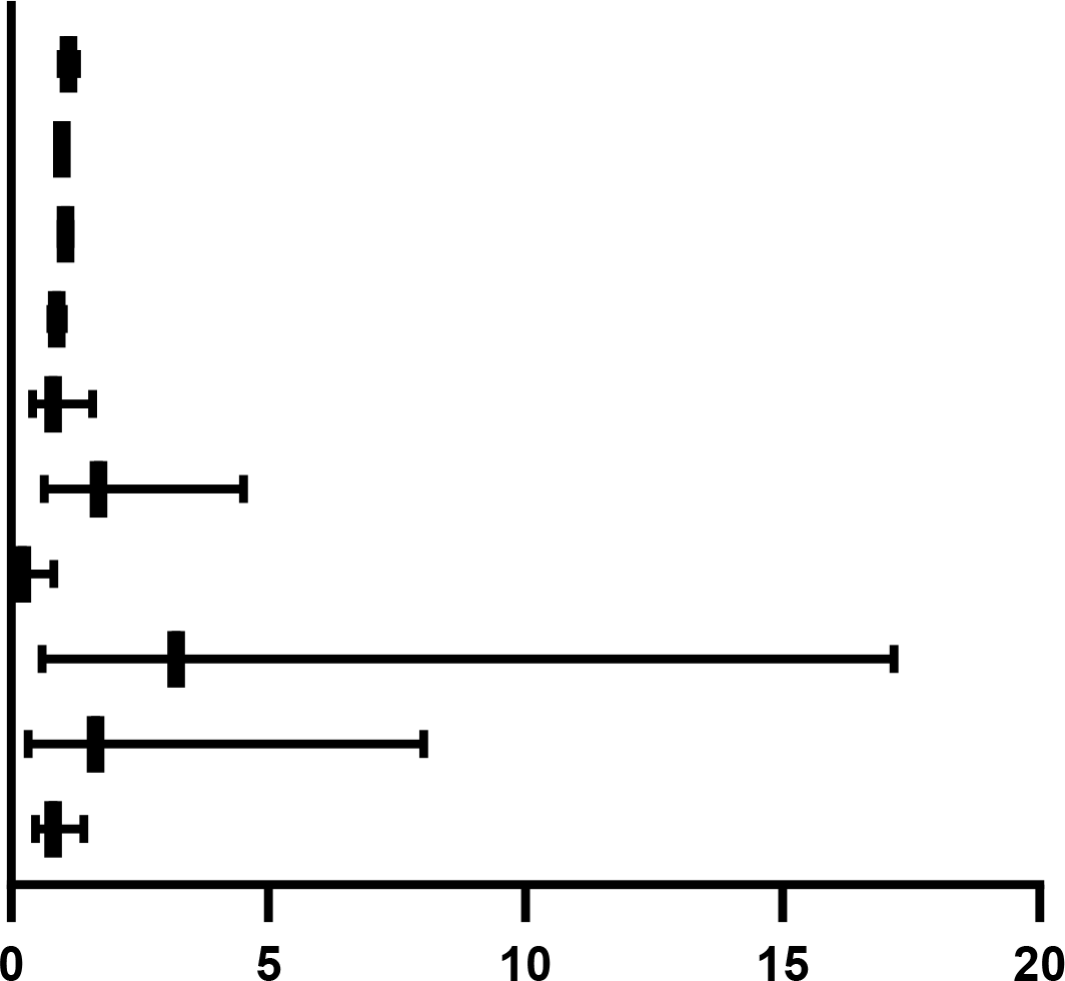
	FT4	0.806	0.992(0.931,1.057)	
	TGAb	0.143	1.058(0.981,1.140)	
	TRAb	0.091	0.892(0.782,1.018)	
	(T0) Nasal reflux score	0.556	0.820(0.424,1.586)	
	(T0) Dysphagia score	0.284	1.706(0.643,4.526)	
	(T0) Dysarthria score	0.027	0.216(0.056,0.836)	
	(T0) Dyspnea score	0.172	3.215(0.602,17.169)	
	(T0) Gag reflex score	0.536	1.648(0.338,8.029)	
	(T0) Muscle weakness score	0.472	0.819(0.475,1.412)	
Cured (T28)	FT3	0.227	1.055(0.967,1.150)	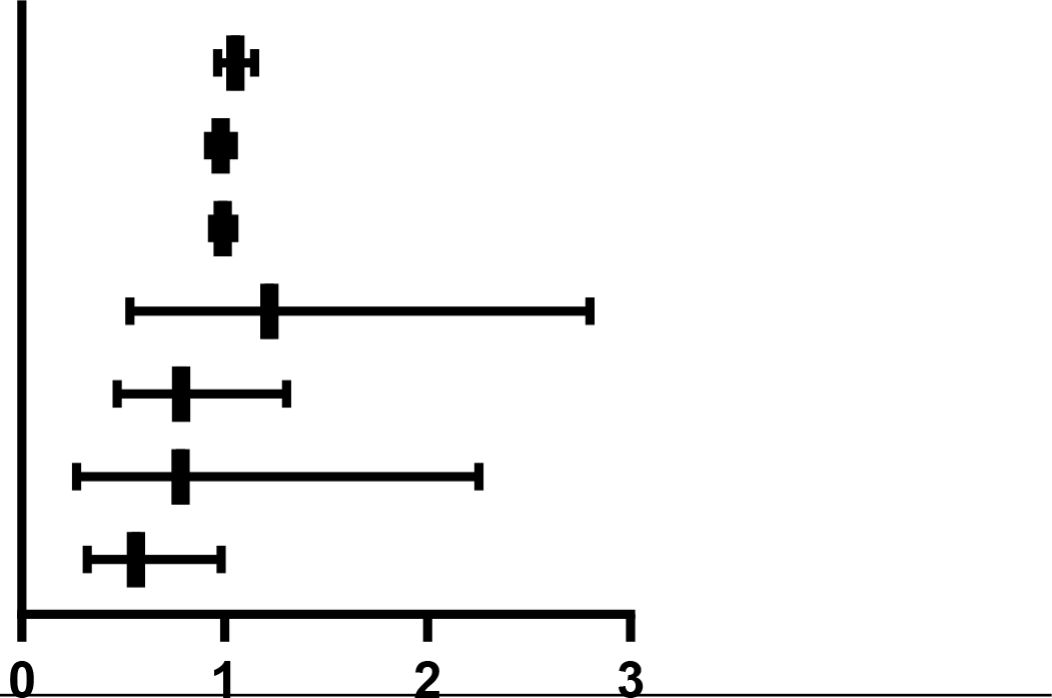
	FT4	0.557	0.982(0.922,1.044)	
	TRAb	0.772	0.992(0.941,1.046)	
	(T7) Dysphagia score	0.636	1.222(0.533,2.801)	
	(T7) Dysarthria score	0.351	0.785(0.472,1.306)	
	(T7) Gag reflex score	0.651	0.783(0.272,2.253)	
	(T7) Muscle weakness score	0.044	0.564(0.323,0.984)	

## 4 Discussion

This study aim to systematically evaluate the clinical efficacy of GCs, ATP and ATDs in treating ATM. The research results indicate that this combination therapy can significantly improve the symptoms of bulbar paralysis and muscle weakness in patients with ATM, showing both sustained and significant efficacy at T7 and T28 follow-up. Notably, this study provides a systematic clinical evaluation of ATM treatment based on a large sample size collected domestically and internationally. Moreover, the evaluation scale used is original and provides important evidence-based support for the comprehensive diagnosis and treatment of ATM.

Since Waldenström first described a group of cases with acute muscle weakness, particularly involving the medullary muscles (12), ATM has gradually been recognized. As clinical experience has grown, a consensus has recognized ATM as a rare but serious complication of thyrotoxicosis, marked by an insidious onset, non-specific symptoms, and high mortality. The disease primarily manifests as pharyngeal muscle weakness, accompanied by difficulties in mastication, swallowing, phonation, and limb weakness, which can potentially progress to lethargy or even coma (2). However, with improvements in medical practice and increased experience in early recognition and diagnosis of ATM, patients with hyperthyroidism presenting with bulbar paralysis and muscle weakness symptoms are increasingly common in clinical diagnosis and treatment. Although some patients present with sudden onset, their severity may be milder than that reported in early cases. After treatment with GCs, ATP and ATDs, symptoms can be relieved or even completely resolved. This improvement indicates that current clinical recognition of ATM may be insufficient, leading to potential missed diagnoses.

The pathogenesis of ATM is not yet clear, and Graves’ disease is one of its most common causes. The core pathogenesis of Graves’ disease involves antigen-presenting cells (such as B cells, macrophages, dendritic cells, and thyroid cells) recognizing and presenting thyroid stimulating hormone receptor (TSHR) peptide segments. These cells activate T cells via MHC molecules and stimulate B cells through the CD40/CD40L pathway. The activated B cells then differentiate into plasma cells and produce TRAb, which promote thyroid cell proliferation and ultimately lead to hyperthyroidism (13). It is worth noting that TSHR is also expressed in neural tissues. TSAb can bind to TSHR expressed on microglial cells, induce their transformation toward the M1 phenotype, activate the NF-κB pathway, and trigger the release of pro-inflammatory factors (8), which may be one of the important pathogenic mechanisms underlying ATM-related medullary paralysis symptoms. We found that the serum CD40 level in ATM patients was significantly higher than that in Graves’ disease patients (14). This finding further supports that the pathogenesis of ATM is closely related to autoimmunity and inflammation. GCs are effective therapeutic drugs for various autoimmune diseases. We speculate that GCs can effectively control the symptoms of bulbar paralysis in ATM patients. The possible mechanisms include: First, GCs enhance the anti-hyperthyroid effect by reducing thyroid hormone secretion and inhibiting the peripheral conversion of thyroxine (T4) to triiodothyronine (T3). A randomized controlled study showed that in patients with type II amiodarone-induced thyroid toxicity, prednisolone restored thyroid function faster than iodothyronine; dexamethasone can reduce serum T3 levels within 24 hours after the first administration (15). Second, GCs exert basic anti-inflammatory effects by directly inhibiting various pro-inflammatory mediators such as CD40, NF-κB, IL-2, IL-6, and TNF-α (16–18). Third, GCs have strong immunosuppressive effects. The key role of antigen-presenting cells (APCs) is to recognize pathogens and present antigen structures to the adaptive immune system, and dendritic cells are the most effective APCs in the innate human immune system. GCs can significantly reduce the number of dendritic cells, suppress the proliferation of induced T cells (CD4+, CD45 RA+) and cytotoxic T cells (CD11-, CD8+), decrease autoantibody production, and thus exert immunosuppressive effects (19).

The pathogenesis of ATM involves central nervous system inflammation and autoimmune mechanisms. It may also be related to energy metabolism disorders caused by high metabolic status and enhanced beta adrenergic activity in the bod (20). Therefore, combined ATP therapy is particularly important. ATP is the main energy carrier within cells and is crucial for maintaining muscle contraction and cellular function. The core mechanism of ATP improving myopathy may include several aspects. Firstly, ATP provides energy to maintain muscle function. Muscle contraction depends on the interaction between myosin and actin, which requires ATP hydrolysis to provide energy (21). Energy metabolism disorders in ATM patients’ skeletal muscles impair muscle function. Thus, ATP supplementation may enhance muscle performance. Secondly, ATP regulates mitochondrial function and oxidative stress. Mitochondria are the main site of ATP synthesis, and their dysfunction can lead to oxidative stress and mitochondrial myopathy, including proximal myopathy. ATP supplementation may alleviate myopathy symptoms by restoring mitochondrial function and reducing the accumulation of reactive oxygen species (22). Thirdly, ATP inhibits the aggregation of pathological proteins. For example, in other neuromuscular disorders, ATP suppresses pathological proteins such as amyloid and alpha synuclein, thereby delaying disease progression of neuromuscular (23–25).

Although there are no guidelines or consensus for ATM treatment, ATDs remain its cornerstone therapy. TH regulates skeletal muscle energy metabolism, activates mitochondrial oxidative pathways, and increases the maximum oxygen consumption of skeletal muscle (26). Moreover, TH is associated with changes in metabolites regulating skeletal muscle energy metabolism, such as acylcarnitine, which affects muscle function recovery in patients with hyperthyroidism (26). High levels of TH can also increase protein energy consumption and reduce protein synthesis, exacerbating muscle weakness in Graves’ disease patients (27). The effect of TH on neuromuscular synapses may also lead to temporary dysfunction of neuromuscular junctions (28). Beyond its impact on limb muscle strength, TH also affects respiratory muscles. Patients with hyperthyroidism have lower respiratory muscle strength and muscle mass than healthy individuals (29).

It is worth noting that ATM patients exhibit more pronounced metabolic hyperactivity and higher circulating TH levels than Graves’ disease patients (11, 14). This may partly explain why ATM patients are more prone to respiratory muscle and limb weakness than those with Graves’ disease alone. Therefore, for ATM patients without contraindications, using ATDs to reduce thyroid hormone levels can effectively improve clinical symptoms of respiratory and skeletal muscle weakness.

However, as an acute complication of hyperthyroidism, ATM cannot be effectively treated with ATDs alone. Previous ATM cases showed that patients treated only with ATDs experienced dysphagia or other bulbar paralysis symptoms, which typically began to improve after 2–3 weeks. For those with more severe conditions, it even took 6–8 weeks to remove the gastric tube (5, 6). When GCs are combined with ATDs treatment, patients often show improvement in dysphagia and dysarthria within a few days to one week (30, 31). In addition, two cases of ATM patients who developed aspiration pneumonia during routine treatment with ATDs and beta-blockers were reported in the literature. The condition only stabilized after treatment with GCs and antibiotics (3, 7). This literature review suggests that although ATDs are an indispensable part of ATM treatment, using ATDs alone is insufficient to effectively control the condition. Therefore, some scholars have proposed that ATM should follow the principles of treating hyperthyroidism crisis and adopt a more proactive comprehensive treatment strategy. This study combined symptomatic treatment using ATDs and beta-blockers with GCs and ATP therapy. As a result, most symptoms improved significantly in the short term. Specifically, within one week, 96.55% of patients experienced relief from dyspnea, 82.76% from rhinitis reflux, and 64% from dysphagia. The average hospitalization lasted about one week. At the T28, over 90% of patients experienced complete relief of the above symptoms, and no patient experienced disease progression or new complications.

Another result of this study showed that ATM patients experience varying recovery times for different symptoms. Symptoms such as dyspnea, rhinitis reflux, and dysphagia improve more rapidly than dysarthria, gag reflex, and muscle weakness. In short-term follow-up, the proportion of dysarthria relief is the lowest, which is a key predictor of short-term efficacy. The one-month follow-up results found that the overall recovery of muscle weakness in ATM patients was slow, which was a key influencing factor for mid-term outcome. Variations in recovery time among symptoms may be related to their location and underlying mechanisms.

The pathogenesis of ATM remains unclear. Several early reports of severe ATM deaths showed pathological changes such as neurodegeneration, hemorrhage, and swelling of oligodendrocytes (10), providing evidence that ATM is a neurological disorder. In clinical practice, Functional Magnetic Resonance Imaging (fMRI) examined the brains of ATM patients, revealing abnormal activity in certain brain regions (32). Symptoms such as dyspnea, nasal reflux, dysphagia, dysarthria, and abnormal pharyngeal reflex have been reported to varying degrees in ATM cases (6, 30, 31). These symptoms closely relate to bulbar lesions, indicating that ATM is likely a disease of bulbar paralysis.

When the pathogenic antibody TRAb binds to nerve cells in the bulbar tissue, it causes immune dysfunction and neuroinflammation. Patients with ATM experience acute edema and inflammatory reactions in the respiratory and pharyngeal muscles, leading to difficulty breathing, swallowing, and nasal reflux. GCs have strong anti-inflammatory and immunomodulatory effects. They rapidly reduce nerve cell inflammation and relieve local spasms and edema in respiratory and pharyngeal muscles, thereby quickly improving airway patency and swallowing function. Excessive TH can directly cause toxic effects on medullary neurons (30), and induce respiratory responses such as hyperventilation, hypoxia, and hypercapnia by increasing central respiratory drive, leading to respiratory muscle weakness and decreased lung capacity (33, 34). Therefore, ATDs reduce TH levels, indirectly alleviating stress damage to respiratory muscles caused by a high metabolic state. This effect partly explains why respiratory distress symptoms in ATM patients improve significantly in the short term.

In contrast, the improvement of symptoms such as dysarthria, delayed pharyngeal reflex, and muscle weakness is slower. A higher baseline dysarthria score is significantly correlated with a lower remission rate at T7, while a higher muscle weakness score at T7 indicates a decreased chance of complete remission at T28. Voice and pharyngeal reflex are regulated by the glossopharyngeal nerve and the paired vagus nerves of the brainstem. Hoarseness and delayed pharyngeal reflex indicate dysfunction of the brainstem or peripheral nerves. Higher scores reflect more severe involvement of the cranial nerves and the pharyngeal muscle groups they innervate, which leads to slower functional recovery.

Limb atrophy and weakness are core manifestations of hyperthyroid myopathy. In this study, 73.81% of ATM patients exhibited limb weakness. After 28 days of treatment, their complete remission rate was lower than that of other bulbar symptoms. The traditional belief is that ATM is caused by the development of CTM, but with deeper understanding, it has been found that ATM can either merge with CTM or appear independently and rapidly deteriorate (2). Based on this, we speculate that in patients with incomplete remission at T28 and combined limb weakness, there is a high possibility of subsequent progression to isolated CTM, but further long-term follow-up and observation are still needed to verify this. Excessive TH promotes cellular metabolism, and the energy required to maintain muscle contraction is consumed in the form of heat production, resulting in insufficient ATP production. At the same time, glycogen breakdown is enhanced and protein synthesis is slowed down, ultimately leading to muscle weakness and atrophy (27). This structural change typically requires a longer repair period, and even if thyroid function returns to normal, complete recovery of neuromuscular function may be delayed. Although the combination therapy of GCs, ATP, and ATDs can regulate the immune system, suppress inflammation, and improve the hypermetabolic state, its ability to reverse existing neuropathy or muscle atrophy is limited. This limitation explains the slow improvement of symptoms like dysarthria, delayed pharyngeal reflex, and muscle weakness.

## 5 Summary

This study found that the combination of GC, ATP and ATDs has significant therapeutic effects on ATM. The observed variability in the recovery of different bulbar paralysis symptoms following combination therapy may be attributed to distinct underlying pathogenic mechanisms. This regimen appears to exert more pronounced effects on immune dysregulation, inflammation, and hypermetabolism than on reversing established neurological damage or muscle atrophy. Clinicians should be aware of the heterogeneity in symptom improvement, particularly in patients with high baseline dysarthria or significant muscle weakness, who may require closer monitoring, personalized treatment adjustments, and proactive metabolic support.

## 6 Limitations

This study is limited by its single-center, retrospective nature, which may incur selection bias. The absence of a concurrent control group treated only with ATDs precludes a direct assessment of the combination therapy’s incremental benefit. Secondly, due to individualized variations in the specific glucocorticoid types administered, the inherent differences in the pharmacokinetics and biological effects of various glucocorticoid formulations might have confounded the precise comparison of efficacy outcomes at T7 and T28. Furthermore, the 28-day follow-up is inadequate for assessing long-term outcomes. Consequently, future research should involve multicenter, prospective designs with longer follow-up, and appropriate control groups and subgroup to validate the regimen’s efficacy and safety profile.

## Data Availability

The original contributions presented in the study are included in the article/supplementary material. Further inquiries can be directed to the corresponding author.
